# Genetic Characterization of Measles and Rubella Viruses Detected Through Global Measles and Rubella Elimination Surveillance, 2016–2018

**DOI:** 10.15585/mmwr.mm6826a3

**Published:** 2019-07-05

**Authors:** Kevin E. Brown, Paul A. Rota, James L. Goodson, David Williams, Emily Abernathy, Makoto Takeda, Mick N. Mulders

**Affiliations:** ^1^Virus Reference Department, Public Health England, Colindale-London, United Kingdom; ^2^Division of Viral Diseases, National Center for Immunization and Respiratory Diseases, CDC; ^3^Global Immunization Division, Center for Global Health, CDC; ^4^National Institute of Infectious Diseases, Tokyo, Japan; ^5^Department of Immunization, Vaccines and Biologicals, World Health Organization, Geneva, Switzerland.

All six World Health Organization (WHO) regions have established measles elimination goals, and three regions have a rubella elimination goal. Each region has established a regional verification commission to monitor progress toward measles elimination, rubella elimination, or both, and to provide verification of elimination* (*1*,*2*). To verify elimination, high-quality case-based surveillance is essential, including laboratory confirmation of suspected cases and genotyping of viruses from confirmed cases to track transmission pathways. In 2000, WHO established the Global Measles and Rubella Laboratory Network (GMRLN) to provide high-quality laboratory support for surveillance for measles, rubella, and congenital rubella syndrome (*3*). GMRLN is the largest globally coordinated laboratory network, with 704 laboratories supporting surveillance in 191 countries (*4*). This report updates a previous report and describes the genetic characterization of measles and rubella viruses during 2016–2018 (*5*). The genetic diversity of measles viruses (MeVs) and rubella viruses (RuVs) has decreased globally following implementation of measles and rubella elimination strategies. Among 10,857 MeV sequences reported to the global Measles Nucleotide Surveillance (MeaNS) database during 2016–2018, the number of MeV genotypes detected in ongoing transmission decreased from six in 2016 to four in 2018. Among the 1,296 RuV sequences submitted to the global Rubella Nucleotide Surveillance (RubeNS) database during the same period, the number of RuV genotypes detected decreased from five in 2016 to two in 2018. To strengthen laboratory surveillance for measles and rubella elimination, specimens should be collected from all confirmed cases for genotyping, and sequences from all wild-type measles and rubella viruses should be submitted to MeaNS and RubeNS in a timely manner.

## Laboratory Surveillance for Measles and Rubella Viruses

Countries report data from measles and rubella cases identified through laboratory-supported case-based surveillance systems to WHO. Laboratory testing includes both serologic and molecular confirmation of suspected cases and genetic characterization of viruses from confirmed cases. Participating GMRLN laboratories report MeV and RuV sequence data^†^ from confirmed cases to MeaNS and RubeNS databases, which were initiated in 2005 as a joint project between Public Health England and WHO.^§^ In addition to the reported sequence data from GMRLN, sequences also are downloaded from GenBank, the genetic database maintained by the National Institutes of Health.^¶^ To ensure the quality of sequence information, GMRLN has established a molecular proficiency testing program and has accredited 86 laboratories within the six WHO regions for MeV and RuV detection and genotyping (*6*).

According to the monthly reports of 184 countries that reported measles and rubella case-based surveillance data in 2018, a total of 317,445 serum specimens were received by the participating GMRLN laboratories from patients with suspected cases, an increase of 101% compared with the number of specimens received in 2016. Among 275,020 (87%) specimens tested for measles immunoglobulin M, 78,950 (29%) were positive; 203,898 (64%) also were tested for rubella immunoglobulin M, and 11,874 (6%) were positive. By the end of 2018, MeaNS contained 47,521 MeV sequences, a 93% increase from the 24,571 sequences reported as of July 1, 2015 (*5*). During this time, the number of RuV sequences in RubeNS increased 73%, from 1,820 to 3,149.

## Characterization of Measles and Rubella Viruses

In addition to monitoring the occurrence and distribution of MeV and RuV genotypes, the characterization of individual circulating wild-type MeVs is critical for monitoring progress toward regional elimination goals. One element of the evidence required for the verification of measles elimination is documentation of ≥12 months with no circulation of an endemic lineage of MeV in the presence of a well-performing surveillance system; verification of measles elimination is achieved after ≥36 months of interrupted measles transmission (*7*). To describe transmission patterns of defined lineages of MeV, GMRLN established standard methods for naming the genetic characteristics of wild-type MeVs derived from the 450 nucleotides sequence encoding the 150 carboxy-terminal amino acids of the N protein (N450), a highly variable region of the genome, including a convention for nominating specific N450 sequences as “named strains” (*5*). Each N450 sequence submitted to MeaNS is assigned a distinct sequence identifier (DSId), allowing viruses with identical N450 sequences to be identified. An index for the diversity of each MeV genotype reported to MeaNS, defined as the number of distinct sequences divided by the total number of records in the database, is calculated. If multiple MeV cases (generally ≥50) with the same DSId are associated with extensive transmission in multiple countries, and if the sequence has been made publicly available by submission to GenBank, then members of GMRLN can request that the N450 sequence be nominated as a named strain. Generally, the name assigned is the WHO name of the earliest example of the strain within MeaNS and does not imply any epidemiologic significance regarding the source of infection.

During 2016–2018, six of the 24 recognized MeV genotypes were detected (Figure). The number of MeV genotypes detected decreased from six (B3, D4, D5, D8, D9, and H1) in 2016 to four (B3, D4, D8, and H1) in 2018 (Table 1). The number of reported cases of MeV genotype H1, which is endemic in China, declined 87%, from 2,625 in 2016 to 333; in 2018, genotypes B3 and D8 accounted for 95% of reported sequences.

**FIGURE:**
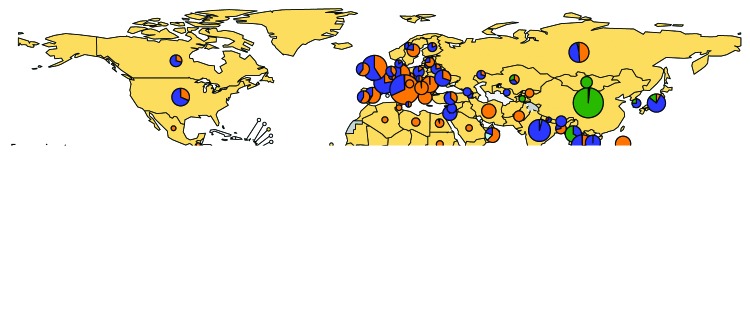
Global distribution of measles virus genotypes,* 2016–2018 * The size of the circles reflects the numbers of replicates reported for each genotype.

**Source:** World Health Organization

**TABLE 1:** Measles virus genotypes, distinct N450* sequences, diversity index,† and rubella virus genotypes reported globally — Measles Nucleotide Surveillance (MeaNS) database and Rubella Nucleotide Surveillance database, 2016–2018

Genotype	2016			2017			2018		
	No. of records (%)	No. of DSIds	Diversity index	No. of records (%)	No. of DSIds	Diversity index	No. of records (%)	No. of DSIds	Diversity index
**Measles virus**									
B3	705 (14)	96	0.136	2,665 (45)	170	0.064	2,923 (44)	219	0.075
D4	51 (1)	7	0.137	15 (<1)	6	0.400	19 (<1)	2	0.105
D5	1 (<1)	1	1.000	N/D	N/D	N/D	N/D	N/D	N/D
D8	1,541 (31)	166	0.108	2,561 (44)	208	0.081	3,396 (51)	281	0.083
D9	96 (2)	11	0.115	46 (<1)	5	0.109	N/D	N/D	N/D
H1	2,625 (52)	204	0.078	544 (9)	70	0.129	333 (5)	40	0.120
**Total**	**5,019 (100)**	**485**	**N/A**	**5,831 (100)**	**459**	**N/A**	**6,671 (100)**	**542**	**N/A**
**Rubella virus**									
1E	10 (4)	N/A	N/A	13 (7)	N/A	N/A	933 (88)	N/A	N/A
1G	6 (3)	N/A	N/A	2 (1)	N/A	N/A	N/D	N/A	N/A
1H	1 (<1)	N/A	N/A	1 (<1)	N/A	N/A	N/D	N/A	N/A
1J	1 (<1)	N/A	N/A	N/D	N/A	N/A	N/D	N/A	N/A
2B	221 (92)	N/A	N/A	172 (91)	N/A	N/A	130 (12)	N/A	N/A
**Total**	**239 (100)**	**N/A**	**N/A**	**188 (100)**	**N/A**	**N/A**	**1,063 (100)**	**N/A**	**N/A**

**Abbreviations**: DSIds = distinct sequence identifiers; N/A = not applicable; N/D = genotype not detected.

* N450: Sequences for the 450-nucleotide carboxy-terminal of the nucleocapsid gene in the measles virus genome. Data from the MeaNS database is available at http://www.who-measles.org/Public/Web_Front/main.php.

^†^ The diversity index for each measles virus genotype reported to MeaNs is defined as the number of distinct sequence identifiers divided by the total number of records.

Also, during 2016–2018, the diversity index decreased for each detected genotype, except for genotype H1, as the number of circulating genotype H1 viruses decreased by 87%. During 2016–2018, 32 named strains were identified (five for genotype B3, 11 for genotype D4, eight for genotype D8, two for genotype D9, and six for genotype H1). Among the 10 most commonly reported named strains, two appeared in all six regions (Table 2).

**TABLE 2:** The 10 most common distinct N450* measles virus (MeV) sequences (named strains) reported globally — Measles Nucleotide Surveillance (MeaNS) database, 2016–2018

DSId*	MeV genotype	MeV strain name	No. of records	No. of countries	No. of WHO regions
4299	B3	MVs/Dublin.IRL/8.16/	2,719	43	4
4221	D8	MVs/Osaka.JPN/29.15/	1,235	32	6
2668	H1	MVs/Hong Kong.CHN/49.12/	1,149	9	4
4807	D8	MVs/Herborn.DEU/05.17/	900	15	3
4683	D8	MVs/Gir Somnath.IND/42.16/	814	36	4
5096	B3	MVs/Saint Denis.FRA/36.17	567	18	3
4283	D8	MVs/Cambridge.GBR/5.16/	561	20	3
2283	D8	MVi/Hulu Langat.MYS/26.11/	494	30	6
2728	H1	MVs/Aichi.JPN/9.13/	388	3	2
4742	D8	MVs/Samut Sakhon.THA/49.16	355	20	4

**Abbreviations**: DSId = distinct sequence identifier; WHO = World Health Organization.

* N450: sequences for the 450-nucleotide carboxy-terminal of the nucleocapsid gene in the MeV genome. Data from the MeaNS database is available at http://www.who-measles.org/Public/Web_Front/main.php.

During 2016–2018, five of the 13 recognized RuV genotypes were detected, and the number of detected RuV genotypes decreased from five in 2016 (58% of the sequences belonged to genotype 1E and 40% to genotype 2B) to two (1E and 2B) in 2018 (Table 1). However, global virologic surveillance for rubella is incomplete. With the exception of the Region of the Americas, which has eliminated rubella, the virus remains endemic in all regions. Among 866 sequences reported to RubeNS in 2018, 837 (96.6%) came from the Western Pacific Region (primarily from China and Japan); the African and Eastern Mediterranean regions, two regions with large numbers of reported confirmed rubella cases, were not represented in the RubeNS database in 2018.

## Discussion

GMRLN continues to provide high-quality laboratory support to surveillance for measles and rubella virus transmission and critical evidence needed for the verification of elimination. The increase in serologic testing and the number of sequences reported to the databases reflect an expansion of the capacity of GMRLN as well as the resurgence of measles in many countries during 2018. With support of the molecular surveillance data provided by GMRLN, measles elimination has been verified by 81 (42%) of the 194 WHO member countries and rubella by 76 (39%) of the 194 countries.** Moreover, the decreasing diversity indices for the most frequently detected MeV genotypes suggest that the number of chains of transmission is decreasing globally because of increasing population immunity. However, many countries reporting laboratory-confirmed measles and rubella cases have failed to collect specimens for genetic characterization, particularly during outbreaks. With only four remaining MeV genotypes detected in circulation and a decrease in sequence variability within MeV genotypes, increases in specimen collection and reporting of sequences to MeaNS from countries with confirmed measles cases are needed to better track MeV transmission patterns. In addition, most countries still have not submitted sufficient sequence information to provide adequate baseline genetic characterization of RuVs.

The MeaNS database recognizes distinct N450 sequences and assigns DSIds to enable the identification of related MeVs in different countries and regions. In addition, a convention of naming the MeV strains with the same DSId is used. However, when defining endemic circulation of a specific MeV strain, caution should be exercised in interpreting the significance of MeV N450 sequences with different DSIds, named strains, or both. Given the conserved nature of the MeV genome, even within the highly variable N450 coding region, identical N450 sequences can be detected over multiple years and thus might not be linked or in the same direct line of transmission within a country or region. Conversely, sequences with a single nucleotide difference within an identified short chain of MeV transmission will be given different DSIds, with different names, even though they might be epidemiologically linked.

The current naming convention does not describe MeV lineages derived from sequence analysis of regions of the MeV genome other than N450. To further differentiate viral transmission chains, additional sequence information from other regions of the genome is needed. Using an expanded sequence window in addition to the N450 sequence has been proposed for countries and regions where measles has been eliminated or is nearing elimination (*8*). To improve the utility of these expanded sequence windows, Public Health England is developing updated versions of the MeaNS and RubeNS databases, along with analysis tools that should be available by the end of 2019. Distinct lineages within RuV genotypes have been described (*9*); however, WHO has not yet recommended a nomenclature for describing these lineages.

The findings in this report are subject to at least two limitations. First, sequences representing chains of transmission in countries with inadequate virologic surveillance are not represented in the global databases. Second, the geographical distribution of sequences reported to the global databases does not align with the distribution of reported measles and rubella cases.

To provide a more comprehensive overview of circulating viruses and their temporal and geographic distribution, strengthening of case-based surveillance by national programs is essential. WHO’s Manual for the Laboratory-based Surveillance of Measles, Rubella, and Congenital Rubella Syndrome provides guidance for increasing specimen collection for virus detection and sequencing (*6*). Countries moving toward elimination are recommended to obtain genotype information from ≥80% of all chains of transmission (i.e., outbreaks or case clusters) (*6*). Once identified by national or regional GMRLN laboratories, all sequences from wild-type MeVs should be submitted to MeaNS and RuVs to RubeNS within 2 months of specimen receipt in the laboratory. Sequences reported in countries should be linked to named strains if possible. When feasible, supplementary information (e.g., travel history, source of infection, and location) should be submitted with sequence information. With increased sequence reporting and use of new sequencing approaches, GMRLN will provide enhanced support for monitoring progress toward and verifying achievement of measles and rubella elimination.

**Summary**

**What is already known about this topic?**

Monitoring progress toward measles and rubella elimination requires high-quality case-based surveillance, including genetic characterization of measles viruses and rubella viruses.

**What is added by this report?**

During 2016–2018, the number of reported measles virus genotypes declined from six to four; two (B3 and D8) accounted for 95% of reported sequences. Of 13 rubella virus genotypes, reported genotypes declined from five to two.

**What are the implications for public health practice?**

Diversity of measles and rubella viruses has decreased globally, consistent with progress toward elimination. Continued collection of specimens from all confirmed cases for genotyping and submission of wild-type virus sequences to global databases will strengthen case-based surveillance.

## Footnotes

* Verification of elimination is defined as the absence of endemic virus transmission for a continuous period of ≥36 months in the presence of a high-quality surveillance system and confirmed by the regional verification commission.

† The standard sequence window for measles virus is the 450-nucleotide carboxy-terminal of the nucleocapsid gene in the MeV genome; the standard sequence window for rubella virus is a 739-nucleotide fragment (nucleotides 8,731–9,469) in the E1 gene in the RuV genome.

§ http://www.who-measles.org/Public/Web_Front/main.php.

¶ https://www.ncbi.nlm.nih.gov/genbank/.

** WHO Immunization, Vaccines and Biologicals database.
